# Heterologous Expression and Characterization of Collagenases from *Pseudomonas chlororaphis* GP72

**DOI:** 10.3390/biology15030247

**Published:** 2026-01-29

**Authors:** Dingkang Hu, Shengjie Yue, Yongkang Huang, Shengxiao Zhang, Chuxuan Gong, Ruxiang Deng, Yanfang Nie, Hongbo Hu, Wei Wang, Xuehong Zhang

**Affiliations:** 1 State Key Laboratory of Microbial Metabolism, School of Life Sciences and Biotechnology, Shanghai Jiao Tong University, Shanghai 200240, Chinaxuehzhang@sjtu.edu.cn (X.Z.); 2 National Experimental Teaching Center for Life Sciences and Biotechnology, Shanghai Jiao Tong University, Shanghai 200240, China

**Keywords:** collagenase, *Pseudomonas chlororaphis*, genetic engineering, heterologous expression, *Escherichia coli*

## Abstract

Collagenases can specifically degrade collagen, showing a wide application prospect in food, leather, waste utilization, biotechnology, and other industries. Currently, *Hathewaya histolytica* is commonly used in industry to produce collagenases, but its application is greatly limited by its pathogenicity. This study first identified five potential *Pseudomonas*-derived collagenases by sequence alignment. Bioinformatics tools were used to analyze their structures and functions. Heterologous expression of two *P. chlororaphis*-derived collagenases was achieved in *E. coli*, and their enzymatic properties were characterized. Bioinformatics analysis shows that the *Pseudomonas*-derived collagenases had low molecular weights (22.1~50.5 kDa) and good thermal stability (aliphatic index 73.73~88.81). Deletion of *P. chlororaphis* GP72ANO strain *colP1* and *colP2* genes had no significant effect on cell growth. The yields of collagenase ColP1 and ColP2 obtained from *E. coli* BL21(DE3) cultivation broth were 148 mg/L and 322 mg/L, respectively. The optimum temperature of each collagenase was 28 °C, and the soluble collagen activities of ColP1 and ColP2 were up to 42.64 U/mg and 21.21 U/mg, respectively. Collagenase ColP1 had the highest enzyme activity at pH 8, while collagenase ColP2 had the highest enzyme activity at pH 4. Metal ions such as Na^+^, K^+^, Mg^2+^, Ca^2+^, Ni^2+^, and Mn^2+^ inhibited the activity of collagenases to different degrees. This study successfully achieved recombinant expression and preliminary purification of *Pseudomonas*-derived collagenases in *E. coli* and explored their function and physicochemical properties.

## Simple Summary

Two novel collagenases from *Pseudomonas chlororaphis* were identified and characterized in this study. Their properties were initially analyzed through bioinformatics, and then the two collagenases were successfully expressed in *Escherichia coli*. Experimental investigations revealed their enzymatic activities under varying temperature conditions, pH levels, and metal ions. Notably, these collagenases may demonstrate distinct advantages in industrial applications due to their compact molecular structure and reduced structural complexity compared to conventional counterparts, suggesting enhanced production feasibility. This work provides new collagenases for industries like food, medicine, and biotechnology, enabling applications such as meat tenderizing and leather processing. In particular, their potential therapeutic applications, such as wound debridement and scar management, position these enzymes as valuable additions to the existing collagenase repertoire.

## 1. Introduction

Collagenases are a class of enzymes that specifically degrade the three-dimensional helix structure of collagen under physiological pH and temperature conditions, which include mammalian matrix metalloproteinases (MMPs), cysteine proteases from mammals, and some proteases from bacteria [1]. Bacterial collagenases mainly include metalloproteases and serine proteases, and the main reported bacterial collagenases are members of the M9 family of the MEROPS database, represented by collagenases from *Hathewaya histolytica* (*Clostridium histolyticum*) and *Clostridium perfringens* [2,3,4]. Some other bacterial collagenases are distributed in the S1, S8, and S53 families [1,4,5,6,7,8,9]. In addition, some members of the U32 family, mainly from pathogenic bacteria, have also been reported to have collagenase activity [1,4,10].

Collagenases are used in the food industry, medical industry, biotechnology industry, and other industrial sectors [11,12,13,14]. Collagenases are also used in the food industry as a meat tenderizer to improve the taste of meat [13]. Their ability to hydrolyze collagen is also used in fur and leather tanning to ensure uniform dyeing of leather [11]. In the medical field, collagenases are applied to treat burns and ulcers, eliminate scar tissue, etc. [15,16].

Currently, anaerobic microorganisms such as *H. histolytica* produce most of the commercially available and more well-studied collagenases. However, *H. histolytica*, as a pathogenic bacterium, may mix toxic and harmful substances such as α-toxin into the product during the collagenase production process [17]. High-purity collagenases require complex purification steps that increase production costs significantly [18]. Other sources of collagenases include *Streptomyces* and kiwifruit; the growth cycle of *Streptomyces* is long, genetic manipulation is difficult, and the extraction of collagenase from kiwifruit is costly and mainly used in the food industry [8,19]. Currently, collagenases have numerous potential applications; however, existing products have limitations that hinder widespread use. The production of collagenases urgently requires safe, efficient, and low-cost methods.

*Pseudomonas* species such as *Pseudomonas aeruginosa* produce a variety of extracellular substances, particularly a variety of proteases [20]. The presence of collagenases in *Pseudomonas* has been identified in early studies, but there are fewer studies related to *Pseudomonas* collagenases [21]. *Pseudomonas* collagenases have a smaller molecular weight than those of *H. histolytica*, and only the active central domain is retained [22,23], making it easier to heterologously express and optimize its properties using genetic engineering.

Currently, it is difficult to obtain *H. histolytica* collagenase that is free of gelatinase and other non-specific proteases [24,25]. Heterogeneity and the difficulty of obtaining mono-isoformic preparations hindered applications of commercial collagenase in biotechnology and medicine [26]. Heterologous expression is an effective way to efficiently produce functional proteins. Currently, heterologous expression of active collagenase from *H. histolytica* in high-efficiency chassis strains *Saccharomyces cerevisiae*, *Bacillus subtilis*, and *Escherichia coli* is reported [26,27,28]. *Pseudomonas* strains lack efficient expression systems, which may lead to difficulties in efficiently producing the target protein. Given that *E. coli* is a well-established platform with a well-defined genetic background and further optimized by engineering protease-deficient strains such as *E. coli* BL21(DE3) [29], it was chosen for collagenase expression in this study.

In this study, bioinformatics methods were employed to screen for collagenases derived from *Pseudomonas*. Heterologous expression of two *P. chlororaphis*-derived collagenases was achieved in *E. coli*. Furthermore, their function and physicochemical properties were explored.

## 2. Materials and Methods

### 2.1. Microorganism, Plasmid, Primers, Media Composition, and Culture Conditions

All of the strains, plasmids, and primers used in this study are listed in Tables S1–S3 [29,30,31,32,33,34,35,36,37].

King’s Broth (KB) medium contains tryptone (20 g/L), glycerol (15 mL/L), K_2_HPO_4_·3H_2_O (0.514 g/L), and MgSO_4_ (0.732 g/L), and GDM medium contains (NH_4_)_2_HPO_4_ (9.9 g/L), K_2_HPO_4_·3H_2_O (7.6 g/L), KH_2_PO_4_ (3.7 g/L), MgSO_4_ (0.12 g/L), glycerol (20 g/L), and FeCl_3_·6H_2_O (3.24 g/L). Luria–Bertani (LB) medium contains tryptone (10 g/L), yeast extract (5 g/L), and sodium chloride (10 g/L), and solid medium contains an additional 2.4 g/L agar.

The *Pseudomonas* strains were cultured in KB medium or GDM medium at 28 °C, and shaken at 200 rpm. For cultivation, *Pseudomonas* strains preserved at −80 °C in a refrigerator were cultured twice at 28 °C for 14 h on LB plates. One single colony of *Pseudomonas* on the LB plate was then activated in 5 mL of the LB liquid medium to serve as a seed culture. Flask cultivation was operated in 250 mL of triplicate baffled flasks containing 60 mL of the KB liquid medium or GDM medium at 28 °C and 200 rpm.

For strain construction and seed culture, *E. coli* strains were grown in LB medium at 37 °C and 200 rpm. For collagenase expression, *E. coli* strains were cultured in 100 mL of LB medium in 500 mL conical flasks at 37 °C for 2 h at 200 rpm, and then turned to 16 °C for 16 h at 150 rpm.

### 2.2. Bioinformatics Approaches

In this study, *Pseudomonas* collagenases were retrieved by BLAST (https://blast.ncbi.nlm.nih.gov/Blast.cgi, accessed on 4 September 2022) from the National Center for Biotechnology Information (NCBI) database in FASTA format [38], the sequences of the proteins were aligned using the multiple sequence alignment clustalW tool in the MEGA11 software (Version 11.0.11), and a phylogenetic tree was constructed using the neighbor-joining method [39,40]. The ProtParam tool (http://web.expasy.org/protparam/, accessed on 13 May 2023), which is commonly used for evaluating the various physicochemical characteristics of proteins, was used to analyze the physicochemical parameters and amino acid composition of the collagenases from the four *Pseudomonas* strains [41]. The structural domains of the studied protein sequences were predicted using the Pfam database (http://pfam.xfam.org/search, accessed on 13 May 2023) [42]. The secondary structures and three-dimensional (3D) model of the studied collagenases were analyzed using Phyre2 and Swiss-Model tools [43,44,45,46,47].

### 2.3. Recombinant Expression Plasmid Construction

The collagenase genes *colP1* and *colP2* were amplified via PCR from *P. chlororaphis* GP72 genomic DNA using *colP1*-F3/*colP1*-R3- and *colP2*-specific primers, respectively. The PCR conditions were as follows: 95 °C for 5 min; 30 cycles of 95 °C for 30 s, 56 °C for 30 s, and 72 °C for 30 s; and then 72 °C for 5 min. The pET28a(+) expression vector was digested with *EcoR* I and *Xho* I, and the amplified *colP1* fragments were ligated into the digested vector using the NovoRec^®^ Plus one-step cloning kit (Novoprotein, Suzhou, China). Ligation products were transformed into *E. coli* DH5α, with positive clones verified by PCR using *colP1*-F3/R3 and confirmed by sequencing (Figure S1a,b). Successfully sequenced plasmids were transformed into protease-deficient *E. coli* BL21(DE3) for expression verification (Figure S2), followed by culture preparation: a single BL21(DE3) colony harboring the recombinant plasmid was inoculated into 5 mL LB medium containing 50 μg/mL kanamycin, cultured at 37 °C with shaking (150 rpm) for 12–16 h, and stored as glycerol stocks at −80 °C. ColP2 recombinant plasmid construction followed identical procedures.

### 2.4. Collagenase Gene Deletion

The *colP1* (*MOK_RS0104145*) and *colP2* (*MOK_RS0102725*) genes were deleted from the *P. chlororaphis* GP72ANO strain using a scarless deletion method. For *colP1* deletion, primers *colP1*-F1 and *colP1*-R1 amplified a 563 bp upstream fragment, while *colP1*-F2 and *colP1*-R2 amplified a 555 bp downstream fragment; these fragments were fused via overlap PCR using primers *colP1*-R1 and *colP1*-F2 (Figure S1c,d). The fused product and pk18mob*sacB* plasmid were digested with *Xba* I and *Hind* III, ligated into the vector using the NovoRec^®^ Plus one-step cloning kit (Novoprotein), and transformed into *E. coli* S17-1 (λpir). The recombinant plasmid was transferred to the GP72ANO strain via conjugation, and double-crossover mutants were screened by colony PCR (Figure S2). Deletion was confirmed by PCR and Sanger sequencing. The *ΔcolP2* mutant was constructed using the same procedure described above.

### 2.5. Recombinant Expression and Purification of Collagenases

For recombinant expression, BL21(DE3) strains preserved at −80 °C in a refrigerator were cultured twice at 37 °C for 14~16 h on an LB plate with 50 μg/mL kanamycin. One colony of BL21(DE3) from the LB plate was then activated into 5 mL of the LB liquid medium to serve as a seed culture with shaking at 37 °C for 12~16 h. Then, 1 mL of bacteria was inoculated into 100 mL LB liquid medium with 50 μg/mL kanamycin and cultured with shaking at 37 °C for 2 h. When the OD_600_ was 0.6, IPTG was added at a final concentration of 0.5 mM and shaken at 16 °C for 16 h. Then, 50 mL of induced bacteria was taken and centrifuged at 5000× *g* for 10 min at 4 °C, and the supernatant was removed. The pellet was resuspended in 5 mL of sterile PBS and centrifuged, and this washing step was repeated twice.

The bacteria pellet was resuspended in 10 mL washing buffer (50 mM Tris-HCl, pH 7.4, 300 mM NaCl, 20 mM imidazole) and subjected to ultrasonic lysis in an ice bath (25% amplitude, 3 s on/8 s off cycles for 20–30 min total). Upon clarification of the bacterial solution, samples were centrifuged at 4 °C and 10,000× *g* for 10 min, and the supernatant and the pellet were collected. The supernatant (9.5 mL) was purified using a His-tagged Protein Purification Kit (CWBIO, Taizhou, China). The target protein was eluted with 5 times the column volume (column volume is 1 mL). Purified recombinant collagenases, pellet, and gradient eluate from the purification process were then analyzed by sodium dodecyl sulfate–polyacrylamide gel electrophoresis (SDS-PAGE).

### 2.6. SDS-PAGE and Western Blot

SDS-PAGE was carried out to determine the successful purification of the recombinant protein and its molecular mass. Coomassie Blue Fast Staining Solution was used for staining the gel, and the molecular mass of collagenases was estimated on a 15% HEPES polyacrylamide gel based on protein standards (TureColor Pre-stained Protein Marker, 2 colors, Wide Range, 10~250 kDa, Sangon Biotech, Shanghai, China).

In order to conduct Western blot, 20 μL protein samples were electrophoresed on sodium dodecyl sulfate–polyacrylamide gel and then transferred to a 0.45 μm polyvinylidene difluoride (PVDF) membrane (Sangon Biotech) by electroblotting. Membranes were blocked with EZ-Buffers J Western Block Buffer in PBS with Non-Fat Milk (Sangon Biotech) at 4 °C for 12 h. After washing with PBS 3 times, the membranes were incubated with Anti-6 × His mouse monoclonal antibody (Sangon Biotech) at 37 °C for 1 h, then washed with PBS 3 times, and incubated with HRP-conjugated Goat Anti-mouse IgG (Sangon Biotech) at 37 °C for 1 h. After washing membranes with PBS 3 times and using Ultra-sensitive horseradish, the catalase DAB color kit (Sangon Biotech) was used in accordance with the manufacturer’s instructions for chemical coloration of proteins, and the blots were then exposed to the camera.

### 2.7. Protein Quantification and Enzyme Assay of Collagenases

The protein concentration was estimated using the Bradford method; the absorbance was measured at 595 nm, and bovine serum albumin was used as the protein standard [48].

Collagenase activity was determined by the colorimetric method. In brief, 80 mg of SnCl_2_·2H_2_O was dissolved in 50 mL of citrate buffer (0.2 M, pH 5.0). Ninhydrin solution was prepared by dissolving 0.5 g of ninhydrin in 10 mL of 2-ethoxyethanol. The ninhydrin reagent for color development was made by mixing SnCl_2_ solution with an equal volume of ninhydrin solution before use. The reaction buffer was composed of 50 mM 2-(tris(hydroxymethyl)methylamino)ethane-1-sulphonic acid (TES), pH 7.4, and 5 mM CaCl_2_. Then, in this assay, 100 μL of enzyme solution and 25 mg of collagen type I or collagen from fish scale and skin were added into 5 mL of reaction buffer and incubated for 3 h at 37 °C. Then the reaction mixture was filtered with a 0.22 μm aqueous phase filter membrane. A 100 μL reaction mixture and 1 mL ninhydrin reagent prepared as described above were added in a 2 mL Eppendorf tube and heated at 100 °C for 20 min in a water bath. Once the tubes are cooled to room temperature, add 1 mL of 50% 1-propanol and mix well. Amino acids (or peptides) resulting from collagen hydrolysis were detected on a Spark multimode microplate reader (Tecan, Männedorf, Switzerland); a sample containing only ninhydrin but without substrate and collagenase in the same volume of reaction buffer was used as a blank, and the absorbance was read at 570 nm. One unit (U) of enzyme activity was defined as the amount of enzyme catalyzing the formation of 1.0 µM L-leucine in 3 h.

When measuring the collagenase activity of fermentation broth, calculate the equivalent concentration of L-leucine in the reaction solution after digesting the substrate through the standard curve, and then calculate the number of micromoles of L-leucine equivalents produced in the reaction between the collagenase and substrate to determine the collagenase activity of the sample. Inactivated enzyme and substrate background was subtracted from the data to eliminate interference.

Example of enzyme activity per unit volume:

Enzyme activity (U/mL) = L-leucine equivalent millimolar concentration (mM) × 5.1 (Total volume of reaction system, mL)/0.1 (Sample volume added to measure enzyme activity, mL).(1)

### 2.8. Effects of pH, Temperature, and Metal Ions on Collagenase Activity

Collagenase activity was assayed under standard conditions at various temperatures (16~60 °C) to determine the optimal temperature of the recombinant enzyme at pH 7.4. All experiments were performed in triplicate.

For the pH dependence assay, the collagenase activity was measured at 28 °C using buffers at different pH levels, including NaAc-HAc buffer (pH 4.0~6.0), Tris-HCl buffer (pH 7.0~9.0), and carbonate–bicarbonate buffer (pH 10.0).

To analyze the effect of metal ions on the enzyme activity, the enzyme solution was added to the reaction buffer with different metal ions, including 3.6 mM Na^+^, K^+^, Mg^2+^, Ca^2+^, Ni^2+^, and Mn^2+^. No additional metal ions were added to the control group. The activity of the enzyme in each solution was measured at 28 °C and pH 7.4. 

Background signals from inactivated samples processed under identical experimental conditions (temperature, pH, and metal ions) were subtracted from all measurements to eliminate interference from non-specific factors.

### 2.9. Statistical Analysis

All data were analyzed using Microsoft Excel 2019 and GraphPad Prism version 10.6.0. Statistical significance was evaluated using one-way ANOVA followed by Tukey’s multiple comparison test as needed with *p* < 0.05 considered to be statistically significant. Figures were created using GraphPad Prism version 10.6.0 software.

## 3. Results

### 3.1. Bioinformatics Analysis

The potential *Pseudomonas* collagenases identified in the *Pseudomonas* genome database by sequence comparison using BLASTN are shown in Table 1. *Pseudomonas* collagenases that have been reported mainly come from the START and U32 families; therefore, collagenase gene sequences of *P. aeruginosa* and *Porphyromonas gingivalis* were used as queries [23,49]. The amino acid sequences of the collagenases encoded by *PA1579*, *colP1* (*MOK_RS0104145*), *H78_RS09665*, and *KR485_RS10250* were 201–202 amino acid residues in length, while *colP2* (*MOK_RS0102725*) encoded a 445-amino-acid collagenase.

**Figure 1:**
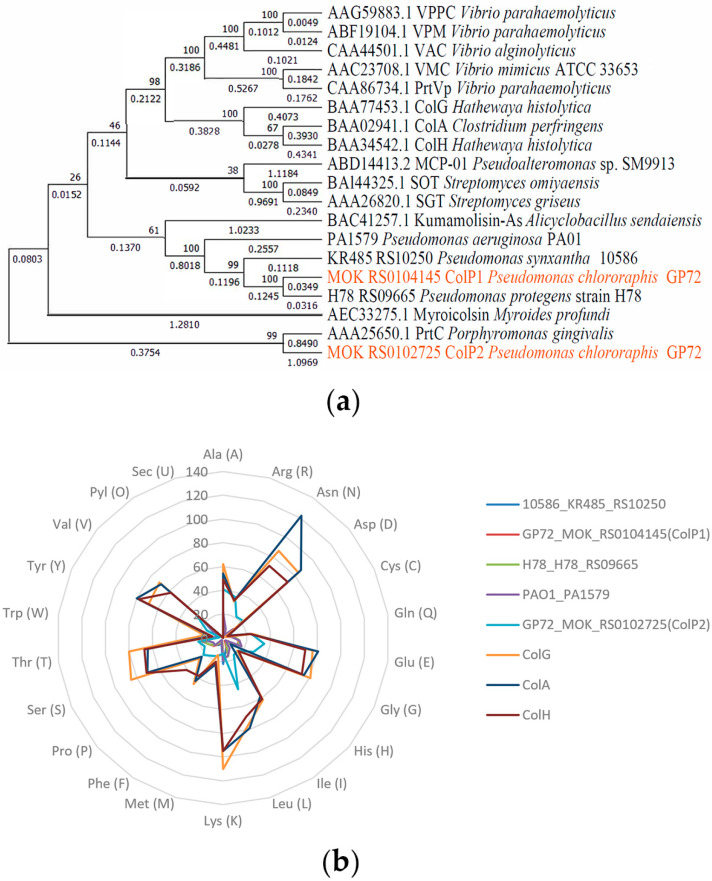
Collagenase phylogeny and amino acid composition analysis. (**a**) Collagenase phylogenetic tree constructed using neighbor-joining method. (**b**) Amino acid composition of collagenases (by number).

Phylogenetic analysis of *Pseudomonas* collagenases revealed that *KR485_RS10250* from *Pseudomonas fluorescens* 10586, *colP1* from *P. chlororaphis* GP72, *H78_RS09665* from *Pseudomonas protegens* H78, and *PA1579* from *P. aeruginosa* PAO1 encoded collagenases that were closely related to the S53 family Kumamolisin-As from *Alicyclobacillus sendaiensis* (Figure 1a). In contrast, the collagenase ColP2 from *P. chlororaphis* GP72 exhibited a closer evolutionary relationship to the U32 family PrtC of *P. gingivalis*, with an evolutionary distance of 1.9459.

The physicochemical properties of the studied collagenases, including the theoretical isoelectric point (pI), molecular weight, aliphatic index, extinction coefficient, and instability index, are summarized in Table 1. Collagenases encoded by *colP1*, *H78_RS09665*, *KR485_RS10250*, and *PA1579* exhibited a molecular weight of approximately 22.1 kDa, whereas ColP2 had a higher molecular weight of 50.5 kDa. The predicted theoretical pI of *Pseudomonas*-derived collagenases ranged from 6.08 to 8.62, and all were higher than those of *H. histolytica* and *C. perfringens* except ColP2, which had a weakly acidic pI of 6.08, while the other four displayed weakly basic pIs. This pI information informs ion exchange chromatography-based purification strategies. The aliphatic index reflects the relative volume occupied by the aliphatic amino acid side chains of globular proteins, which can indicate hydrophobic interactions, one of the main driving forces in protein folding, and usually, the higher the aliphatic index, the better the thermal stability of the protein [41]. The extinction coefficient of proteins can reflect the absorption of light at a certain wavelength; the extinction coefficients of all five collagenases in this study were lower than those from *H. histolytica* and *C. perfringens*. The instability index can be used to measure the stability of a protein in vitro. Among the five collagenases studied, the instability coefficients were less than 40, except for the collagenase ColP2.

**Figure 2:**
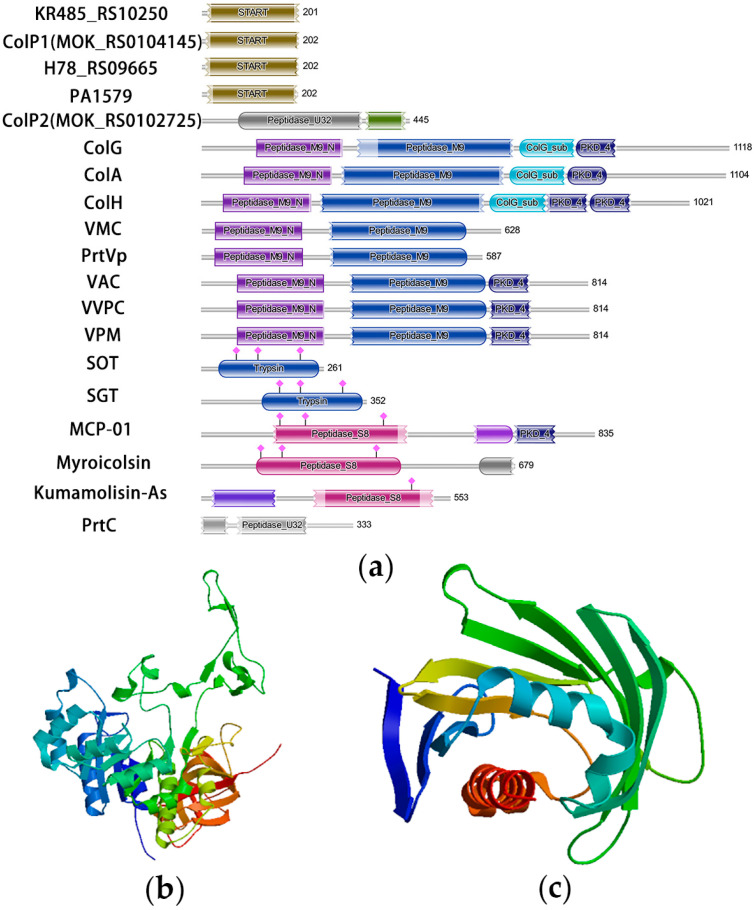
Analysis of domain and three-dimensional structure of collagenases predicted by Swiss-Model. (**a**) Analysis of collagenase domain. (**b**) Three-dimensional structure prediction of collagenase ColP1. (**c**) Three-dimensional structure prediction of collagenase ColP2.

A radar chart comparing the amino acid composition of eight collagenases is presented in Figure 1b, showing the number of amino acids in different collagenases. The domains of the studied collagenases and some known collagenases were predicted by the Pfam tool [42], and the results are shown in Figure 2a. Based on domain analysis, collagenases encoded by *KR485_RS10250*, *colP1*, *H78_RS09665*, and *PA1579* possess unique domain architectures distinct from most previously characterized collagenases, whereas ColP2 showed similarity to U32 family collagenases. Secondary structures of ColP1 and ColP2 were predicted using Phyre2 (Figures S3 and S4). The three-dimensional models of collagenases were predicted by Swiss-Model homology modeling [43]: ColP1 (Figure 2b) utilized a *Xanthomonas axonopodis* STAR-associated lipid transfer protein (template 3qsz.1.A) with GMQE 0.76 and 39.89% sequence identity, while ColP2 (Figure 2c) employed an AlphaFold-predicted model (A0A1Q8EMP5_9PSED from *P. chlororaphis*) with superior quality metrics (GMQE 0.83, 95.72% sequence consistency). The predicted collagenase protein models are all monomers in the oligomeric state.

### 3.2. Expression of Recombinant Collagenases

**Figure 3:**
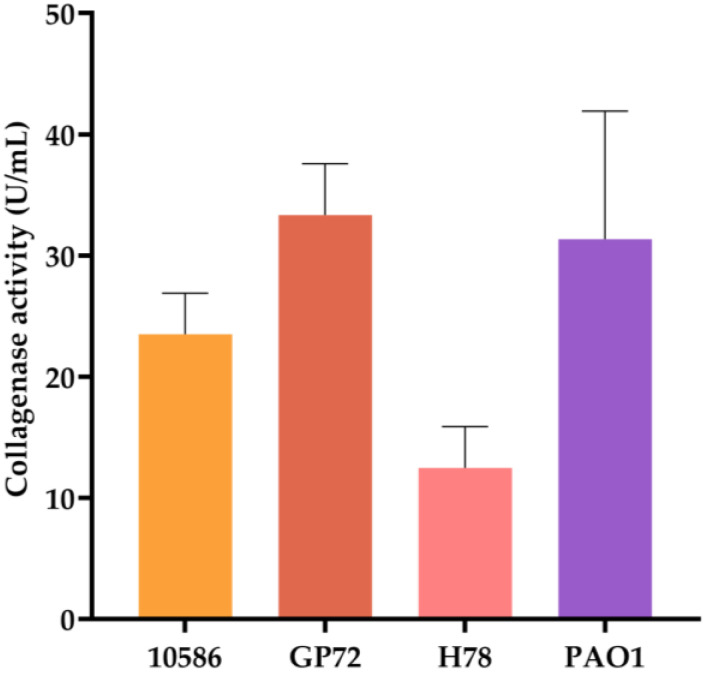
Comparison of collagenase activity in culture supernatants of different strains. Error bars represent standard deviation.

Type I collagen was assessed as the substrate to compare the collagenase activity of different *Pseudomonas* strains. Previous studies have reported that the fermentation supernatant of *P*. *aeruginosa* PAO1 can promote collagen degradation in a concentration-dependent manner [50]. Therefore, PAO1 can be used as a positive control with collagenase activity. Culture supernatants were collected 24 h after inoculation, and collagen degradation was measured after subtracting the background from blank medium controls. As shown in Figure 3, supernatant from GP72 exhibited the highest collagenase activity at 33.35 U/mL, indicating that GP72 produces substantial amounts of collagenase. For heterologous expression, *E. coli* BL21(DE3) was selected over *P. chlororaphis* due to its highly efficient T7 expression system and genetic modifications (knockout of *lon* protease and outer membrane protease *OmpT*), which minimize degradation of expressed proteins [29]. This strain was chosen to express two GP72-derived collagenases.

**Figure 4:**
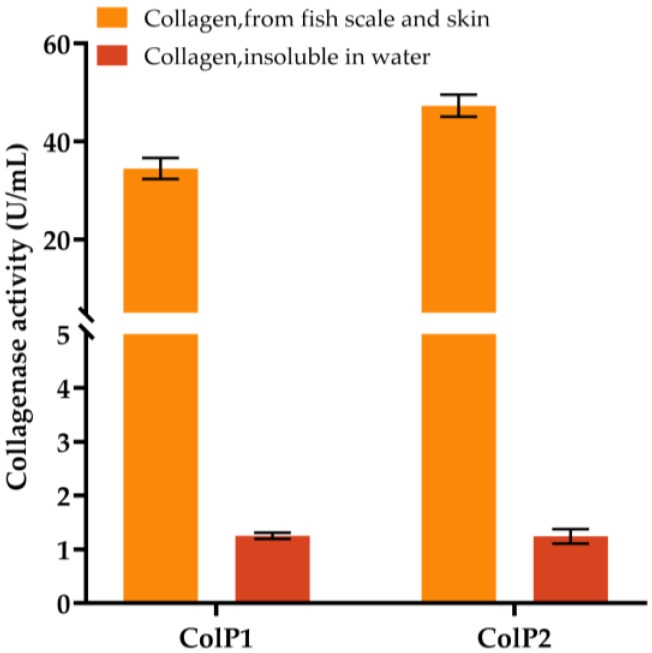
Enzyme activity assay of soluble collagen from fish scales and skin and insoluble collagen using crude enzyme obtained by heterologous expression. Error bars represent standard deviation.

Heterologous expression strains of *E. coli* BL21(DE3) were constructed, and the relative molecular masses of the recombinant proteins were predicted to be 22.1 kDa and 50.5 kDa. The expression of collagenases was induced by *E. coli* BL21(DE3) with pET28a(+)-*colP1*, and BL21(DE3) with pET28a(+)-*colP2* for 16 h at 150 rpm and 16 °C. SDS-PAGE analysis of cell lysates showed protein bands at around 22.1 kDa and 50.5 kDa, confirming the expression of collagenases, which were not present in the negative control samples. The collagenase activity was initially determined using insoluble collagen and soluble collagen from fish scales and skin, respectively, as substrates, and the blank control was protein purification buffer. The results are shown in Figure 4. Enzyme activity assays demonstrated that both collagenases degraded soluble collagen (from fish scale and skin) more efficiently than insoluble type I collagen, with ColP1 and ColP2 reaching activities of 34.48 U/mL and 47.30 U/mL (soluble) versus 1.25 U/mL and 1.24 U/mL (insoluble), respectively. These results confirm the collagen-degrading capability of recombinant collagenases. Due to higher activity and superior experimental controllability, soluble collagen was selected as the substrate for subsequent enzyme characterization.

### 3.3. Purification of Recombinant Collagenases

**Figure 5:**
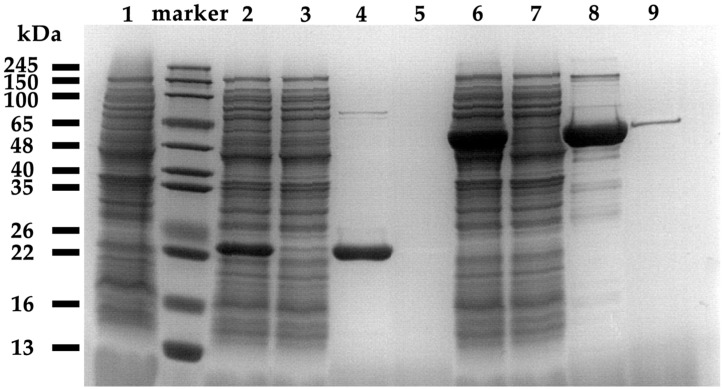
Recombinant expression and purification of protein SDS-PAGE result. Lane 1 is the supernatant of blank control broken cells, Lanes 2~5 are collagenase ColP1 broken cell products, Lanes 6~9 are collagenase ColP2 broken cell products, Lanes 2 and 6 are centrifuged supernatants of broken cells, Lanes 3 and 7 are flow-through fluids, Lanes 4 and 8 are elution peaks, and Lanes 5 and 9 are eluents after elution peaks.

The recombinant collagenases were purified via Ni-affinity chromatography after cell lysis by sonication and centrifugation. SDS-PAGE and Western blot (Figure 5 and Figures S5–S7) confirmed soluble protein bands at ~22 kDa and ~48 kDa—consistent with theoretical masses of 22.1 kDa (ColP1) and 50.5 kDa (ColP2). The protein ColP1 appeared in the 50 mM imidazole elution fractions and then appeared in a large number of fractions of 100 mM and 250 mM elution fractions, and ColP2 appeared in the 50 mM imidazole elution fractions. The protein ColP2 began to appear in large quantities in the 250 mM imidazole elution fraction.

During the purification process, 5 mL of the target protein solution was purified from the 9.5 mL supernatant obtained by centrifugation after ultrasonic disruption of the cells. By calculating the total enzyme activity of the supernatant collected after sonication and the purified enzyme solution on soluble collagen at 37 °C, the yields of ColP1 and ColP2 were 14.5% and 17.1%, respectively. The concentration of the purified protein solution was determined by the Bradford method, and 2.15 mg ColP1 and 5.5 mg ColP2 were obtained from 5 mL protein solution. The yields of collagenases ColP1 and ColP2 obtained from *E. coli* BL21(DE3) cultivation broth were 148 mg/L and 322 mg/L, respectively. The purified recombinant collagenases were subsequently employed for further characterization.

### 3.4. Effect of pH, Temperature, and Metal Ions on the Activity of Collagenases

**Figure 6:**
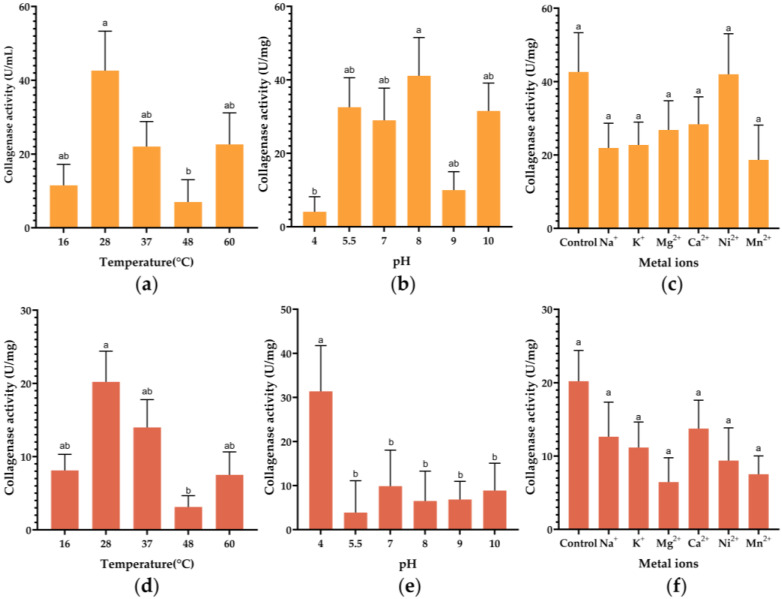
Effect of pH, temperature, and metal ions on the activity of collagenases. (**a**) Effect of temperature on ColP1 collagenase activity; (**b**) effect of pH change on ColP1 collagenase activity; (**c**) effect of metal ions on ColP1 collagenase activity. (**d**) Effect of temperature on ColP2 collagenase activity; (**e**) effect of pH change on ColP2 collagenase activity; (**f**) effect of metal ions on ColP2 collagenase activity. Different letters indicate that values are significantly different (*p* < 0.05). Bars represent standard errors. Orange represents ColP1 and brick red represents ColP2.

The initial purified collagenase samples were reacted with the substrates at temperature gradients of 16 °C, 28 °C, 37 °C, 48 °C, and 60 °C, and the results of collagenase activity measurements are shown in Figure 6a,d. Properties of collagenases were determined by using the soluble collagen from fish scales and skin as the substrate. The enzyme activity increased with increasing temperature when the reaction temperature was lower than 28 °C, consistent with the optimal growth temperature of *P. chlororaphis*. Enzyme activity increased with rising temperatures up to 28 °C (reaching 42.64 U/mg for ColP1 and 20.21 U/mg for ColP2) and subsequently declined at higher temperatures (37–60 °C). This thermal profile confirms 28 °C as the optimal temperature for both enzymes.

The preliminary purified collagenase samples were incubated at different pHs at 28 °C, and the results of the enzyme activity measurements are shown in Figure 6b,e. Results demonstrated that ColP1 exhibited peak enzymatic activity of 41.12 U/mg at pH 8, while ColP2 reached maximum activity of 31.39 U/mg at pH 4, indicating distinct optimal pH profiles for these two collagenase variants.

The results of the recombinant collagenase activity assay under the influence of different metal ions, Na^+^, K^+^, Mg^2+^, Ca^2+^, Ni^2+^, and Mn^2+^, are shown in Figure 6c,f. For ColP1, Ni^2+^ maintained near-control activity (42.00 U/mg vs. control 42.64 U/mg), while other ions (Na^+^, K^+^, Mg^2+^, Mn^2+^) reduced activity. In contrast, ColP2 exhibited significantly decreased activity with Ca^2+^ (13.75 U/mg vs. control 20.21 U/mg), and all other tested metal ions further suppressed enzymatic activity compared to their respective controls.

### 3.5. Effects of Knockout of Collagenase Genes on Cell Growth

To investigate the effect of *colP1* and *colP2* knockout on the growth of strain GP72ANO, KB medium and GDM medium were used to evaluate the growth of cells in rich medium and inorganic salt medium after knocking out collagenase genes. The growth curves in KB and GDM medium are shown in Figure S8. Deletion of *colP1* or *colP2* showed no obvious effect on growth, indicating that collagenase is not required for growth under standard conditions.

## 4. Discussion

Collagenase, which specifically degrades collagen, shows broad application prospects in industries such as food, leather, waste utilization, and biotechnology. Currently, *H. histolytica* is commonly used in industry to produce collagenases, but the pathogenicity of the production strain results in high purification costs and limits its application. This study successfully achieved the recombinant expression and preliminary purification of collagenases derived from *P. chlororaphis* in *E. coli*, and explored their function and physicochemical properties.

Bioinformatics tools were used to study the physical and chemical properties and structural functions of *Pseudomonas* collagenases, and a phylogenetic tree was constructed to analyze the evolutionary relationship between various collagenases, showing that the collagenase ColP2 is closely related to the PrtC of the U32 family. This phylogenetic clustering revealed functional evolutionary adaptations. U32 family enzymes are characterized by a rigid β-barrel substrate-binding pocket that facilitates collagen degradation under acidic conditions—consistent with *Streptococcus mutans*’s pathogenic niche in the periodontium [51]. ColP1’s START domain likely enables membrane association and lipid-mediated collagen targeting [52], explaining its weakly basic pI (7.73) and neutral pH activity. ColP1 may represent a novel START-domain adaptation in collagen degradation. The U32 domain of ColP2 may help improve binding to collagen under acidic conditions, which is consistent with the characteristics of its production strain that often appears in acidic environments such as dental caries and its weakly acidic pI (6.08).

The collagenase ColP2 has a molecular weight of about 50.5 kDa, which is smaller than that of those from *H. histolytica* and *C. perfringens.* Pal reported that the molecular weights of collagenases are usually in the range of 39 to 124 kDa [53]. Lower molecular weight may help reduce diffusion barriers in applications such as wound debridement. The extinction coefficients of ColP1 and ColP2 were lower than those of collagenases from *H. histolytica* and *C. perfringens*, and the lower extinction coefficients indicate that the selected proteins have better interactions with water molecules [54]. The low extinction coefficient indicates excellent hydrophilicity, which facilitates stability during product formulation. The instability index can be used to measure the stability of a protein *in vitro*. Usually, proteins with an instability coefficient of less than 40 are considered to be stable [41]. The instability coefficient of ColP1 is less than 40, except for the collagenase ColP2, which had an instability coefficient of greater than 40, indicating better stability. The stability of ColP1 (lability index < 40) makes it suitable for long-term storage in biocatalytic applications, while the instability of ColP2 (index > 40) can be used for controlled pH-triggered degradation in therapeutic environments (e.g., acidic tumor microenvironment).

Collectively, these *Pseudomonas* collagenases represent two evolutionarily distinct classes: ColP2 (U32 family) may be better at functioning in acidic application scenarios, while ColP1 (START domain) enables collagen breakdown under neutral conditions and has better stability. Their unique domain structure, pH-dependent activity profile, and tailored biophysical properties make them excellent candidates for collagenase applications.

The level of collagenase activity is also influenced by the method used to measure its enzymatic activity, including factors such as different substrates, pH, and temperature. Currently, commonly used collagenase activity detection methods include the Rosen assay, synthetic peptides (FALGPA/Pz peptide), and azocoll assay. The Rosen method, based on the ninhydrin reaction, offers advantages in both qualitative and quantitative detection accuracy and reliability. In contrast, methods such as synthetic peptides and the azocoll assay cannot directly reflect the hydrolytic activity of collagenase toward natural collagen [55]. Sigma company defines the enzyme activity of collagenase I as follows: after reacting for 5 h at pH = 7.4 and 37 °C (in the presence of calcium ions), the amount of polypeptide released from collagen is equivalent to the amount of color developed by 1 μmol of leucine and ninhydrin, which is one CDU (one Collagen Digestion Unit). Various methods, such as ammonium sulfate precipitation, ultrafiltration, immobilized metal affinity chromatography, amylose affinity chromatography, gel filtration chromatography, ion exchange chromatography, size exclusion chromatography, and N-terminal tag removal, have been applied to the purification of collagenases [53]. In a purification study of collagenase derived from *H. histolytica*, the use of a His-tag for Ni-affinity chromatography resulted in partial loss of enzyme activity. Therefore, other purification processes need to be used for collagenase derived from *C. histolytica*, such as using ammonium sulfate and Q-Sepharose chromatography [56].

In this study, the yields of collagenase ColP1 and ColP2 obtained from *E. coli* BL21(DE3) cultivation broth were 148 mg/L and 322 mg/L, respectively. ColG and ColH from *H. histolytica* and ColT from *Clostridium tetani* were also heterologously expressed in *E. coli* and achieved >10 mg/L production [26]. Our yield exceeds the threshold of 200 mg/L generally considered economically viable for industrial enzymes. In contrast, the two collagenases in this study achieved higher yields. Analysis of the crude enzyme in the supernatant collected after ultrasonic disruption of cells and centrifugation showed that the enzyme activities of the collagenases ColP1 and ColP2 for soluble collagen from fish scales and skin can reach up to 34.48 U/mL and 47.30 U/mL, respectively. The crude enzyme activity of ColP1 and ColP2 for insoluble type I collagen degradation can, respectively, reach 1.25 U/mL and 1.24 U/mL, indicating that they also have the ability to degrade type I collagen. Previous studies reported that active collagenase can be expressed in both prokaryotic and eukaryotic expression systems. Collagenases from *H. histolytica* were secreted by the yeast *S. cerevisiae*, and recombinant collagenase titers reached 68 U/mL and 55 U/mL for ColG and ColH [27]. ColH from *H. histolytica* was achieved through extracellular expression in *B. subtilis*, and the enzyme activity could reach 669 U/mL [56]. In comparison, the crude enzyme obtained in this study had lower activity in interpreting type I collagen. The commercial collagenase activity currently on the market from *H. histolytica* is 125 U/mg. In this study, after the two heterologously expressed collagenases were purified by nickel columns, the enzyme activity of ColP1 toward soluble collagen could reach 42.64 U/mg, and the enzyme activity of ColP2 toward soluble collagen could reach 20.21 U/mg. The recombinant collagenase ColH expressed in *B. subtilis* was purified through a purification process suitable for industrial production with a specific activity of 565.25 U/mg [56]. Previous studies have also reported collagenase from *Pseudomonas* with collagenase activity of 2.02 U/mg [21]. Chen et al. used the same definition and determination method of collagenase activity as in this study to determine the activity of *Bacillus cereus* MH19 collagenase ColB recombinantly expressed in *E. coli*, and the purified enzyme activity could reach 200.76 U/mg [57]. In a purification study of *H. histolytica* collagenase, a His-tag for Ni-affinity chromatography may result in loss of enzyme activity [56]. This indicates that the collagenase separation and purification process for ColP1 and ColP2 still needs to be further optimized.

The optimal temperature of both collagenases was 28 °C. Most of the previously studied optimal temperatures for collagenase were between 30 °C and 70 °C [58]. Higher enzyme activity at lower temperatures is advantageous for reducing heating costs in industrial applications. The collagenase ColP2 has the highest enzyme activity at pH 4, and the collagenase ColP1 has the highest enzyme activity at pH 8. The well-studied *H. histolytica* collagenase ColG had the highest enzyme activity at pH 7.4 [59], and the optimal pH of most collagenases was between 5 and 9.5 [53].

For the collagenase ColP2, its enzyme activity is similar to that of the control group in the presence of Ca^2+^ ions. The addition of other metal ions will reduce the collagenase activity. The collagenase activity of ColP1 will decrease under the condition of additional metal ions. According to a previous study, Bhattacharya reported that Ca^2+^ plays an important role in the formation of collagen–collagenase–metal complexes by some collagenases, such as some U32 family collagenases [60], but in the present study, Ca^2+^ did not significantly increase the activity of collagenase, and the activity of collagenase ColP1 was also decreased.

This study can help to overcome the limitations of existing collagenases in the application field, expand the application of collagenases in the food industry, pharmaceutical industry, and household chemical industry, and reduce the production cost of collagenases, and is also very important for the ecological development of the leather industry and other related high-pollution industries. In a follow-up study, the collagenases’ structure and catalytic mechanism can be analyzed by X-ray crystal diffraction or Cryo-electron microscopy. The substrate spectrum of *Pseudomonas* collagenase still needs further study. Subsequent studies can use *B. subtilis* and other strains with an efficient secretory expression system to achieve efficient secretory expression of collagenases, so that the cultivation broth can be used as a crude enzyme after simple treatment. Economic optimization of the production and purification process of *Pseudomonas* collagenase and effective cost accounting studies are also key to the commercial application of *Pseudomonas* collagenase.

## 5. Conclusions

In this study, five potential collagenase genes of *Pseudomonas* were obtained from the *Pseudomonas* genome database by homologous sequence comparison. The knockout of genes *colP1* and *colP2* had no significant effect on the growth of cells. The *E. coli* expression vectors pET28a(+)-*colP1* and pET28a(+)-*colP2* were constructed to heterologously express the two collagenases in BL21(DE3). The recombinant collagenases were proven to possess collagenase activity by determining their activity in different temperature, pH, and metal ion conditions, suggesting their preliminary biochemical properties. The yields of collagenases ColP1 and ColP2 obtained from *E. coli* BL21(DE3) cultivation broth were 148 mg/L and 322 mg/L, respectively. The enzyme activity of ColP1 toward soluble collagen could reach 42.64 U/mg, and the enzyme activity of ColP2 toward soluble collagen could reach 20.21 U/mg, showing degradation activity against soluble collagen. ColP1 and ColP2 crude enzymes can, respectively, reach 1.25 U/mL and 1.24 U/mL for insoluble type I collagenase activity. SDS-PAGE yielded bands of the target proteins around 22 kDa and 48 kDa, which indicated that the molecular weights of the recombinant collagenases were close to the theoretical ones. The optimal temperature of both collagenases was 28 °C, and the enzyme ColP1 had the highest enzyme activity at pH 4, while ColP2 had the highest enzyme activity at pH 8. For the collagenase ColP1, the enzyme activity in the presence of Ni^2+^ ions was similar to that of the control group, and the addition of other metal ions decreased the collagenase activity. The collagenase ColP2 showed a decrease in collagenase activity with the addition of metal ions.

## Figures and Tables

**Table 1:** Physicochemical properties of collagenases.

Strains	Locus Tag/Name	Amino Acid Number (aa)	Molecular Weight (Da)	Theoretical pI	Aliphatic Index	Extinction Coefficient	Instability Index
*P. chlororaphis* GP72	*MOK_RS0104145*	202	221,722	7.73	78.17	40,575	22.08
*P. chlororaphis* GP72	*MOK_RS0102725*	445	505,354	6.08	83.10	39,100	46.54
*P. protegens* H78	*H78_RS09665*	202	222,273	7.71	78.22	42,065	27.98
*P. fluorescens* 10586	*KR485_RS10250*	201	221,334	8.62	73.73	38,055	25.69
*P. aeruginosa* PAO1	*PA1579*	202	221,104	7.71	88.81	42,065	30.45
*H. histolytica*	ColG	1118	1,262,417	5.62	72.10	174,435	26.70
*C. perfringens*	ColA	1104	1,261,058	4.89	71.41	179,950	24.38
*H. histolytica*	ColH	1021	1,163,774	5.92	70.93	165,970	35.44

## Data Availability

The raw data supporting the conclusions of this article will be made available by the authors on request.

## Supplementary Materials

The following supporting information can be downloaded at https://www.mdpi.com/article/10.3390/biology15030247/s1. Figure S1: Schematic diagram of pET28a(+) recombinant plasmid and pK18mob*sacB* knockout plasmid used in this study. Figure S2: Schematic diagram of homology double-crossover knockout. Figure S3: The secondary structure of collagenase ColP1. Figure S4: The secondary structure of collagenase ColP2. Figure S5: ColP1 recombinant expression gradient elution. Figure S6: ColP2 recombinant expression gradient elution. Figure S7: Recombinant expression and purification of protein WB result. Figure S8: Growth curves of *colP1* and *colP2* gene knockout strain in KB and GDM medium. Table S1: Strains used in this study. Table S2: Plasmids used in this study. Table S3: Primers used in this study.
