# Supplementary material for: Heterologous Expression and Characterization of Collagenases from Pseudomonas chlororaphis GP72
Source: Biology (Basel). 2026 Jan 29;15(3):247. doi: 10.3390/biology15030247 (PMC12896945; doi:10.3390/biology15030247)
Supplement: Supplementary file 1 [file biology-15-00247-s001.zip › biology-4090164-supplementary materials.pdf]

Supplementary Materials

Heterologous expression and characterization of collagenases from *Pseudomonas chlororaphis* GP72

Dingkang Hu [1], Shengjie Yue [1], Yongkang Huang[1], Shengxiao Zhang[1], Chuxuan Gong[1], Ruxiang Deng[1], Yanfang Nie[1], Wei Wang[1], Xuehong Zhang [1,2], and Hongbo Hu[1, *]

[1] State Key Laboratory of Microbial Metabolism, School of Life Sciences and Biotechnology, Shanghai Jiao Tong University, Shanghai, 200240, China;

[2] National Experimental Teaching Center for Life Sciences and Biotechnology, Shanghai Jiao Tong University, Shanghai, 200240, China;

* Correspondence: hbhu@sjtu.edu.cn (Hongbo Hu)

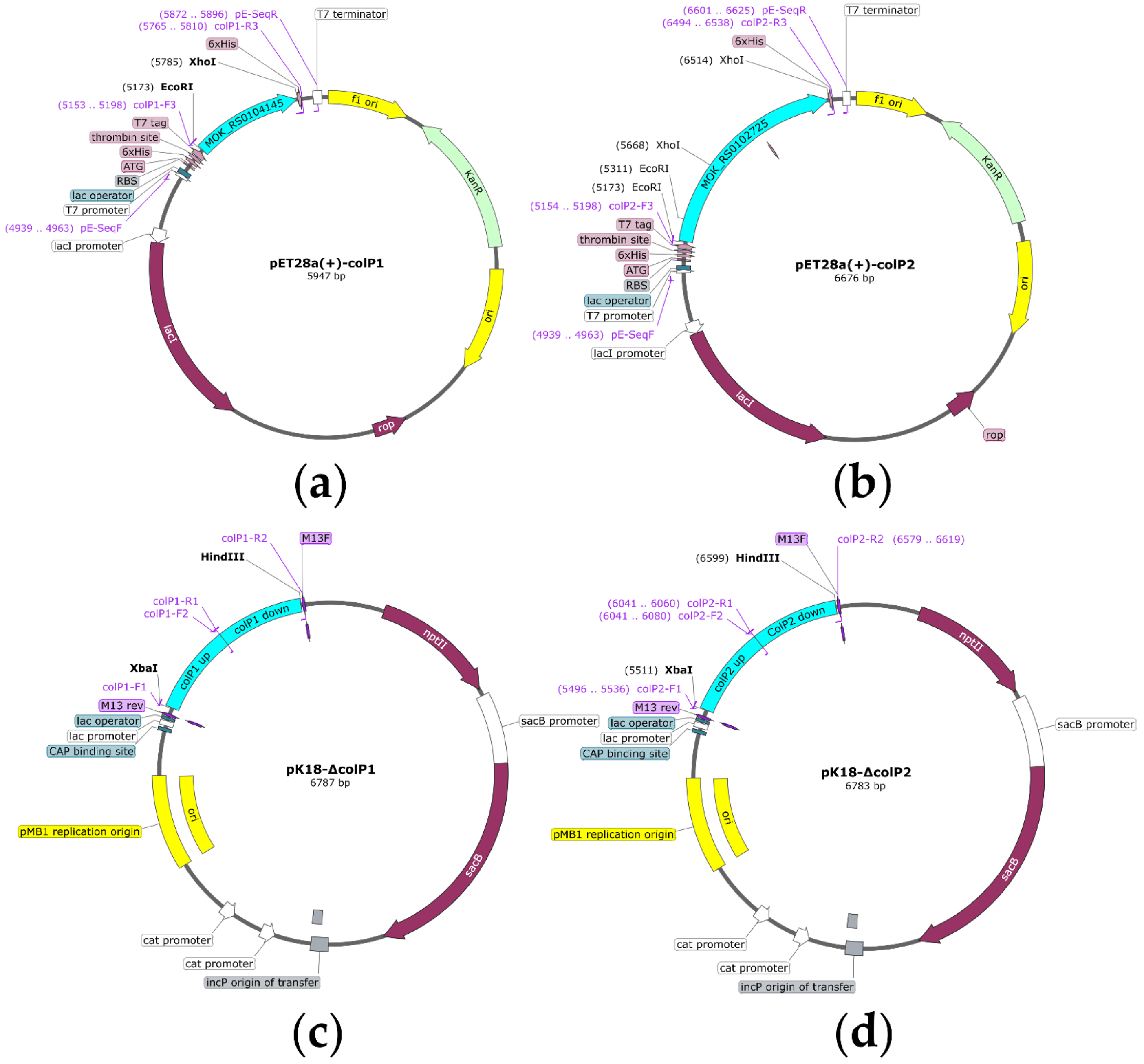

Figure S1. Schematic diagram of pET28a(+) recombinant plasmid and pK18mob*sacB* knockout plasmid used in this study (a) pET28a(+)-*colP1*; (b) pET28a(+)-*colP2*; (c) pK18-Δ*colP1*; (d)pK18-Δ*colP2*.

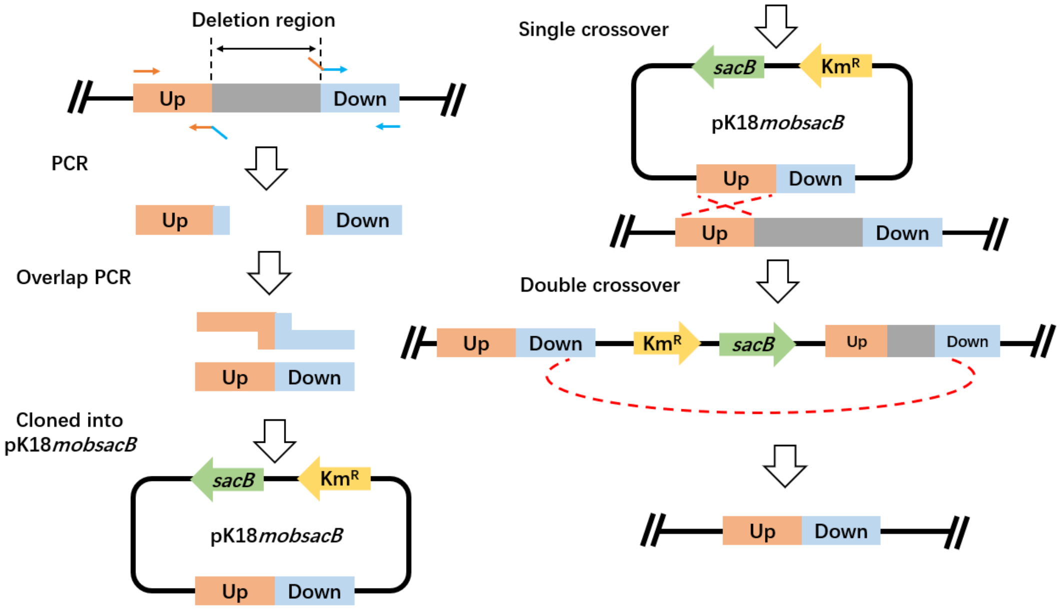

Figure S2. Schematic diagram of homology double crossover knockout. In the first recombination step, the engineered plasmid containing 500–1000 bp DNA fragments upstream and downstream of the target gene is introduced into the host, where it cannot replicate autonomously and thus integrates into the chromosome via a single crossover. In the second step, bacteria are grown under counter-selective conditions (typically on medium with 5%–20% sucrose and no antibiotic) to induce loss of the plasmid.

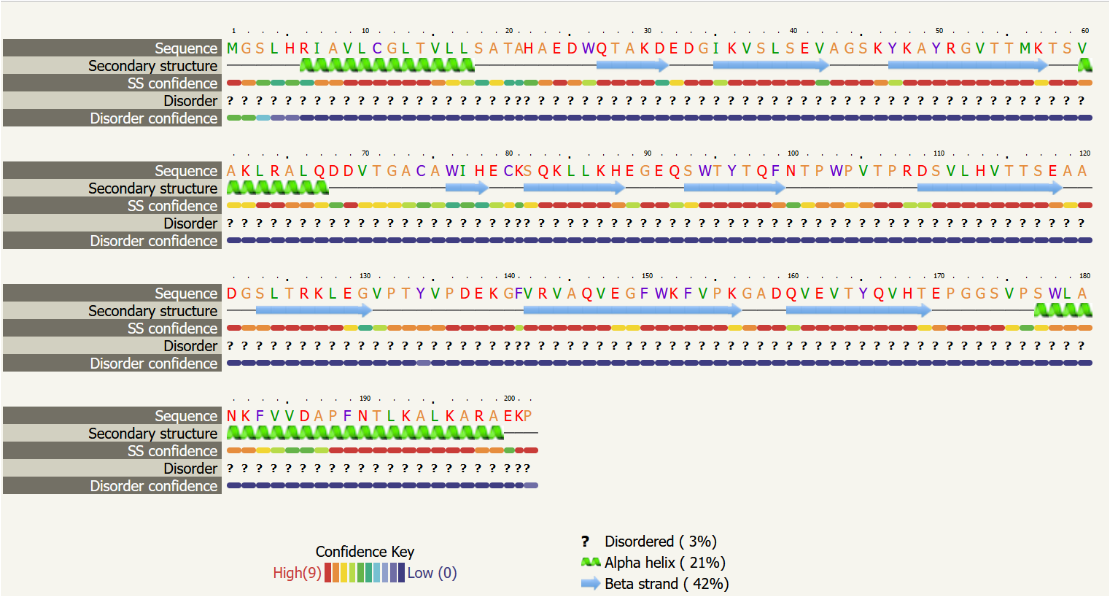

Figure S3. The secondary structure of collagenase ColP1[1]

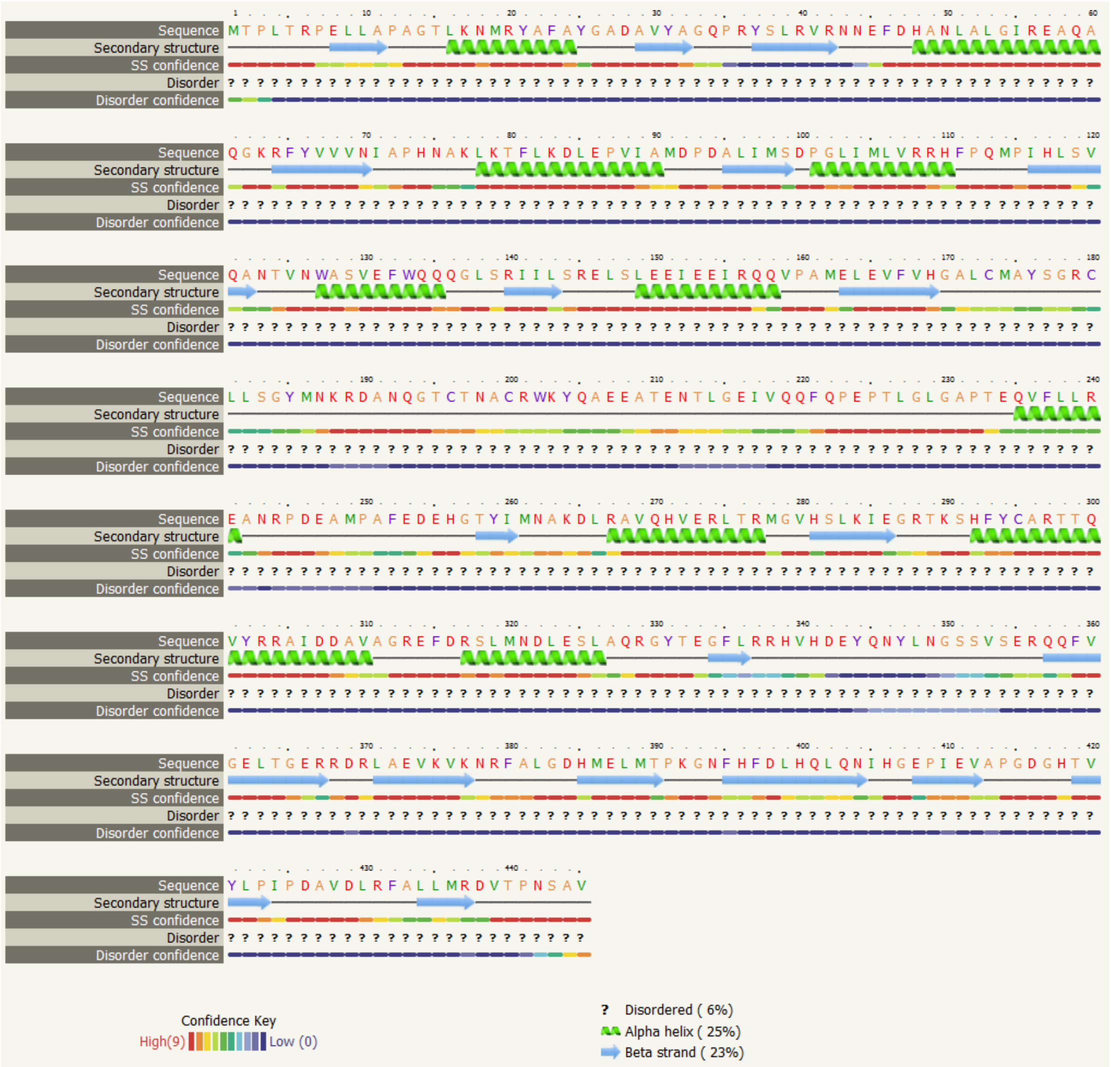

Figure S4. The secondary structure of collagenase ColP2[1]

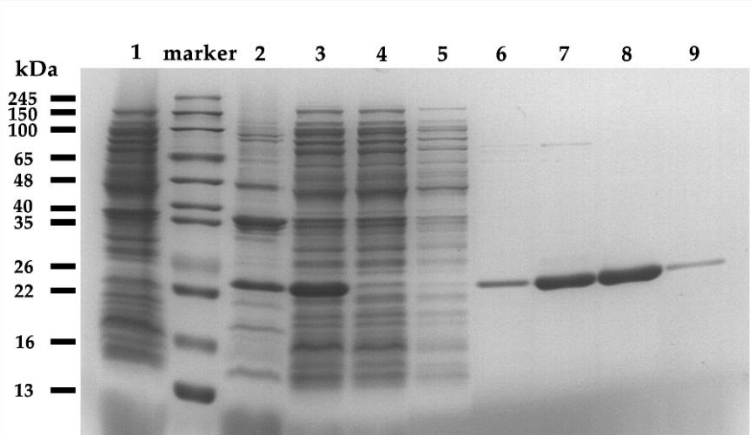

Figure S5. ColP1 recombinant expression gradient elution. Lane 1 is the blank control supernatant of broken cells, lane 2 is the pellet after centrifugation of broken cells (insoluble fractions), lane 3 is the supernatant (soluble fractions), lane 4 is the flow-through fluid, lane 5 is the 20 mM imidazole eluent to elute impurity proteins, and lane 6 ~ 9 is eluted with 50 mM, 100 mM, 250 mM, and 500 mM imidazole eluent in sequence. The theoretical molecular weight of the target protein is 22.2 kDa.

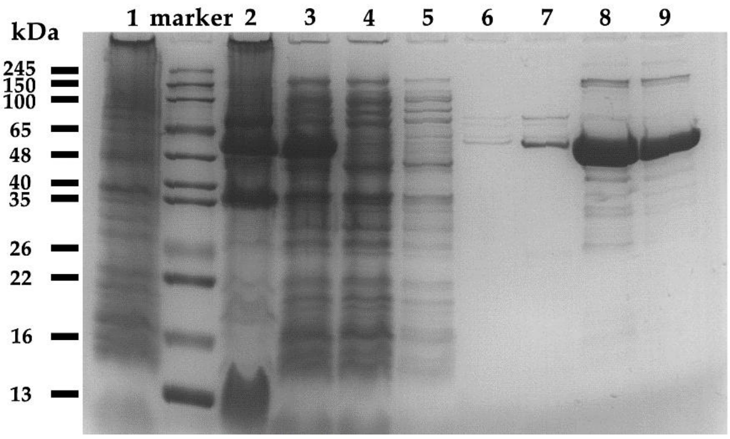

Figure S6. ColP2 recombinant expression gradient elution. Lane 1 is the blank control supernatant of broken cells, lane 2 is the pellet after centrifugation of broken cells (insoluble fractions), lane 3 is the supernatant (soluble fractions), lane 4 is the flow-through fluid, lane 5 is the 20 mM imidazole eluent to elute impurity proteins, and lane 6 ~ 9 is eluted with 50 mM, 100 mM, 250 mM, and 500 mM imidazole eluent in sequence. The theoretical molecular weight of the target protein is 50.5 kDa.

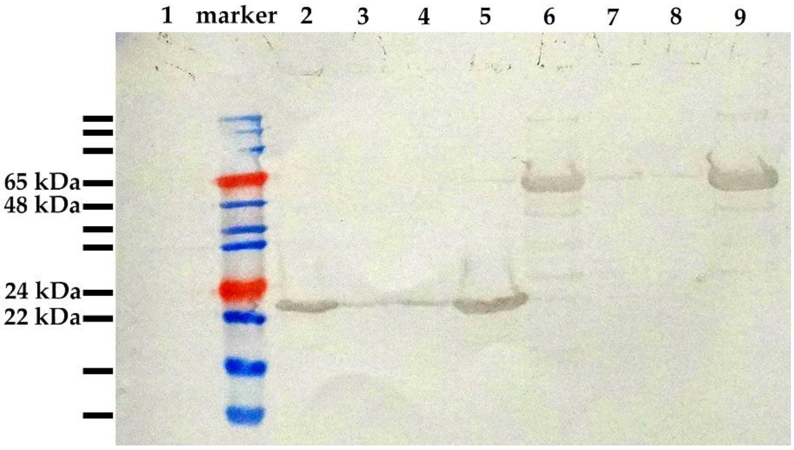

Figure S7. Recombinant expression and purification of protein WB result. Lane 1 is the supernatant of blank control broken cells, Lane 2~5 is collagenase ColP1 broken cell products, Lane 6~9 is ColP2 collagenase broken cell products, Lane 2 and 6 are centrifuged supernatants of broken cells, Lane 3 and 7 are flow-through fluids, Lane 4 and 8 are 50 mM imidazole elution fluids, and Lane 5 and 9 are eluents elution peaks (250 mM imidazole elution fluids).

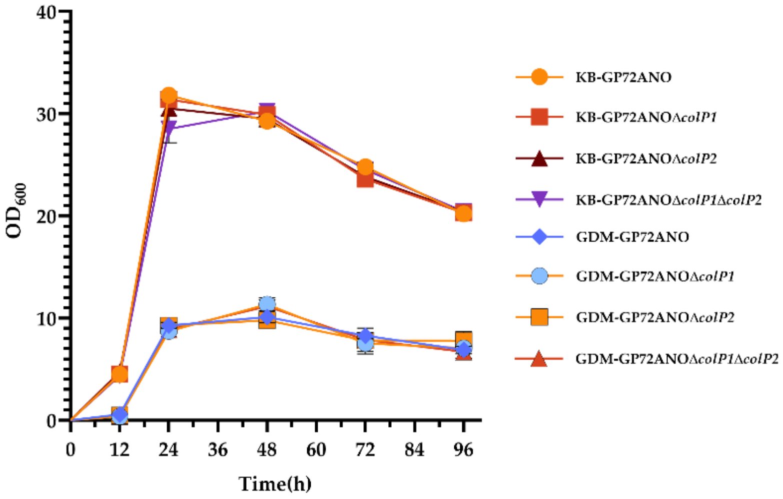

Figure S8. Growth curves of *colP1* and *colP2* gene knockout strain in KB and GDM medium. Bars represent standard errors.

Table S1. Strains used in this study.

Strains	Feature	Source	References
E.coli			
DH5α	*F⁻ φ80lacZΔM15 Δ(lacZYA-argF)U169 recA1 endA1 hsdR17(rK⁻, mK⁺) phoA supE44 λ⁻thi-1 gyrA96 relA1*	store in lab	[2]
BL21(DE3)	*F–ompT hsdSB (rB⁻, mB⁻) gal dcm (DE3)*	store in lab	[3]
P. fluorescens			
10586	*P. fluorescens* 10586	store in lab	[4]
P. chlororaphis			
GP72	*P. chlororaphis* GP72	store in lab	[5]
GP72ANO	*P. chlororaphis* GP72*ΔrpeAΔphzO*	store in lab	[6]
GP72ANO-*ΔcolP1*	*P. chlororaphis* GP72*ΔrpeAΔphzOΔcolP1*	this study	-
GP72ANO-*ΔcolP2*	*P. chlororaphis* GP72*ΔrpeAΔphzOΔcolP2*	this study	-
GP72ANO-*ΔcolP1ΔcolP1*	*P. chlororaphis* GP72*ΔrpeAΔphzOΔcolP1ΔcolP2*	this study	-
P. protegens			
H78	*P. protegens* H78	store in lab	[7]
P. aeruginosa			
PAO1	*P. aeruginosa* PAO1	store in lab	[8]

Table S2. Plasmids used in this study.

Plasmids	Feature	Source	References
pK18mob*sacB*	Broad-host-range gene replacement vector; *sacB*, Km^r	store in lab	[9]
pET28a(+)	expression vector, contains P_{T7} promoter and His-Tag.; Km^r	store in lab	[10]
pET28a(+)-*colP1*	pET28a(+) with *EcoRI- XhoI* insert of 658 bp segments flanking *colP1*, Km^r	this study	-
pK18-*ΔcolP1*	pK18mobsacB with *EcoRI- XbaI* insert of 1118 bp segments flanking *ΔcolP1*, Km^r	this study	-
pK18-*ΔcolP2*	pK18mobsacB with *EcoRI- XbaI* insert of 1092 bp segments flanking *ΔcolP2*, Km^r	this study	-
pET28a(+)-*colP2*	pET28a(+) with *EcoRI- XhoI* insert of 1385 bp segments flanking *colP2*, Km^r	this study	-

Table S3. Primers used in this study.

Primers	Base sequence (5′- 3′)	Source
colP1-F3	AGCAAATGGGTCGCGGATCCGAATTC ATGGGTTCGCTGCATCGAAT	this study
c*olP1*-R3	CAGTGGTGGTGGTGGTGGTGCTCGAG CGGCTTCTCGGCGCGCGCCT	this study
colP2-F3	GCAAATGGGTCGCGGATCCGAATTCA TGACGCCCCTGACCCGCCC	this study
colP2-R3	AGTGGTGGTGGTGGTGGTGCTCGAGC ACCGCGCTGTTCGGCGTCA	this study
*colP*1-F1	CCGGGGATCCTCTAGAAGGCGCGGCG CTGGATTTCG	this study
*colP*1-R1	AGAACGGTCAACCCACAGAG	this study
colP1-F2	CTCTGTGGGTTGACCGTTCTTCAACAC CCTCAAGGCTCTG	this study
colP1-R2	GGCCAGTGCCAAGCTTGCATGTAGCT GACAAGACCA	this study
colP2-F1	GGTACCCGGGGATCCTCTAGATGCTTG AACCGGTCCAGCTG	this study
colP2-R1	GTGCCGGCGGGGGCGAGCAG	this study
colP2-F2	CTGCTCGCCCCCGCCGGCACTTGCCCT GTTGATGCGCGAC	this study
colP2-R2	ACGACGGCCAGTGCCAAGCTTTCAGG GCACTGGCCAGCCGC	this study
PE-SeqF	ACGATGCGTCCGGCGTAGAGGATCG	this study
PE-SeqR	AGGCCCCAAGGGGTTATGCTAGTTA	this study

References

1. Kelley, L.A.; Mezulis, S.; Yates, C.M.; Wass, M.N.; Sternberg, M.J. The Phyre2 web portal for protein modeling, prediction and analysis. *Nature protocols* **2015**, *10*, 845–858.
2. Hanahan, D. Studies on transformation of *Escherichia coli* with plasmids. *Journal of molecular biology* **1983**, *166*, 557–580.
3. Studier, F.W.; Moffatt, B.A. Use of bacteriophage T7 RNA polymerase to direct selective high-level expression of cloned genes. *Journal of molecular biology* **1986**, *189*, 113–130.
4. Cai, Y.; Huang, P.; Venturi, V.; Xiong, R.; Wang, Z.; Wang, W.; Huang, X.; Hu, H.; Zhang, X. Global Gac/Rsm regulatory system activates the biosynthesis of mupirocin by controlling the MupR/I quorum sensing system in *Pseudomonas* sp. NCIMB 10586. *Applied and Environmental Microbiology* **2025**, *91*, e01896–01824.
5. Yue, S.-J.; Li, Y.-X.; Liu, K.-Q.; Zhang, S.-X.; Huang, P.; Wang, W.; Zhang, X.-H.; Hao, X.-R.; Jiang, L.; Hu, H.-B. Efficient bioconversion of corn stover hydrolysates into phenazine-1-carboxylic acid by engineered *Pseudomonas chlororaphis* for sustainable biopesticide production. *Bioresource Technology* **2025**, 133371.
6. Yue, S.-J.; Zhou, Z.; Huang, P.; Wei, Y.-C.; Zhan, S.-X.; Feng, T.-T.; Liu, J.-R.; Sun, H.-C.; Han, W.-S.; Xue, Z.-L. Development of the Static and Dynamic Gene Expression Regulation Toolkit in *Pseudomonas chlororaphis*. *ACS Synthetic Biology* **2024**, *13*, 913–920.
7. Nie, C.; Huang, X.; Xiang, T.; Wang, Z.; Zhang, X. Discovery and characterization of the PpqI/R quorum sensing system activated by GacS/A and Hfq in *Pseudomonas protegens* H78. *Microbiological Research* **2024**, *287*, 127868.
8. Müller, M.M.; Hörmann, B.; Syldatk, C.; Hausmann, R. *Pseudomonas aeruginosa* PAO1 as a model for rhamnolipid production in bioreactor systems. *Applied microbiology and biotechnology* **2010**, *87*, 167–174.
9. Schäfer, A.; Tauch, A.; Jäger, W.; Kalinowski, J.; Thierbach, G.; Pühler, A. Small mobilizable multi-purpose cloning vectors derived from the *Escherichia coli* plasmids pK18 and pK19: selection of defined deletions in the chromosome of *Corynebacterium glutamicum*. *Gene* **1994**, *145*, 69–73.
10. Pan, S.-h.; Malcolm, B.A. Reduced background expression and improved plasmid stability with pET vectors in BL21 (DE3). *Biotechniques* **2000**, *29*, 1234–1238.
