# Supplementary figures and images for: Heterologous Expression and Characterization of Collagenases from Pseudomonas chlororaphis GP72
Source: Biology (Basel). 2026 Jan 29;15(3):247. doi: 10.3390/biology15030247 (PMC12896945; doi:10.3390/biology15030247)

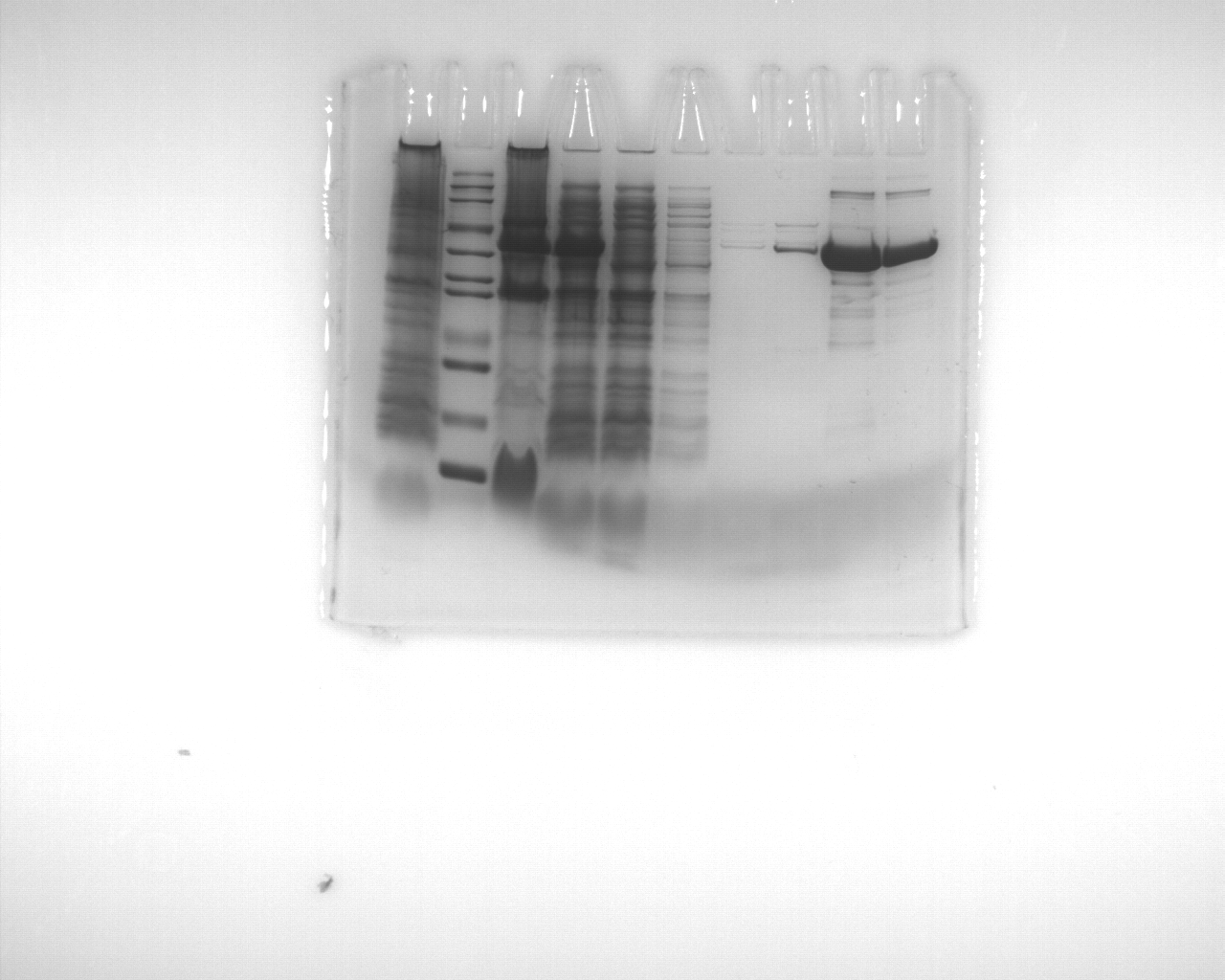

Supplement: Supplementary file 1 [file biology-15-00247-s001.zip › supplementary files - original images/original images of Figrue S6.bmp]

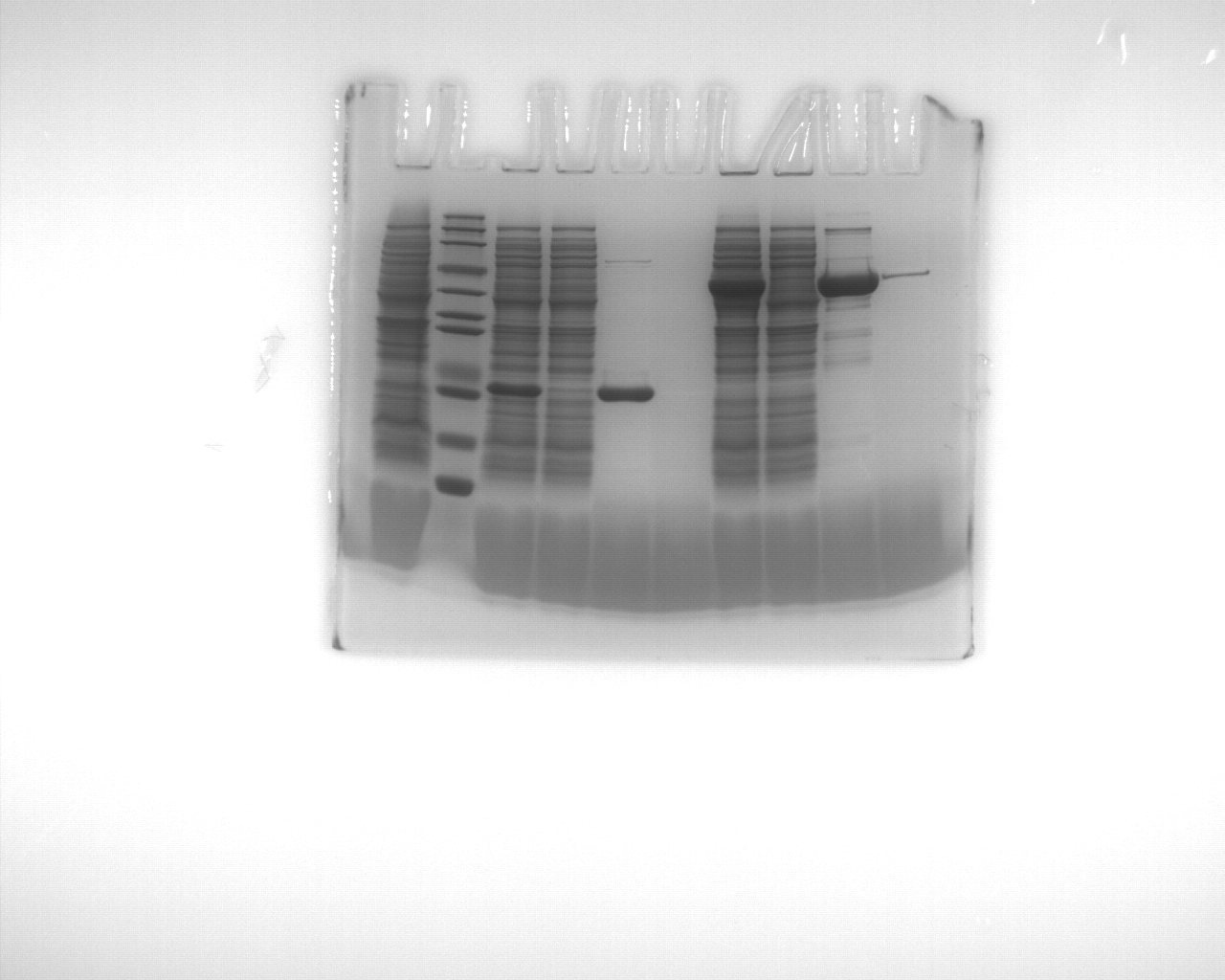

Supplement: Supplementary file 1 [file biology-15-00247-s001.zip › supplementary files - original images/original images of Figure 5.bmp]

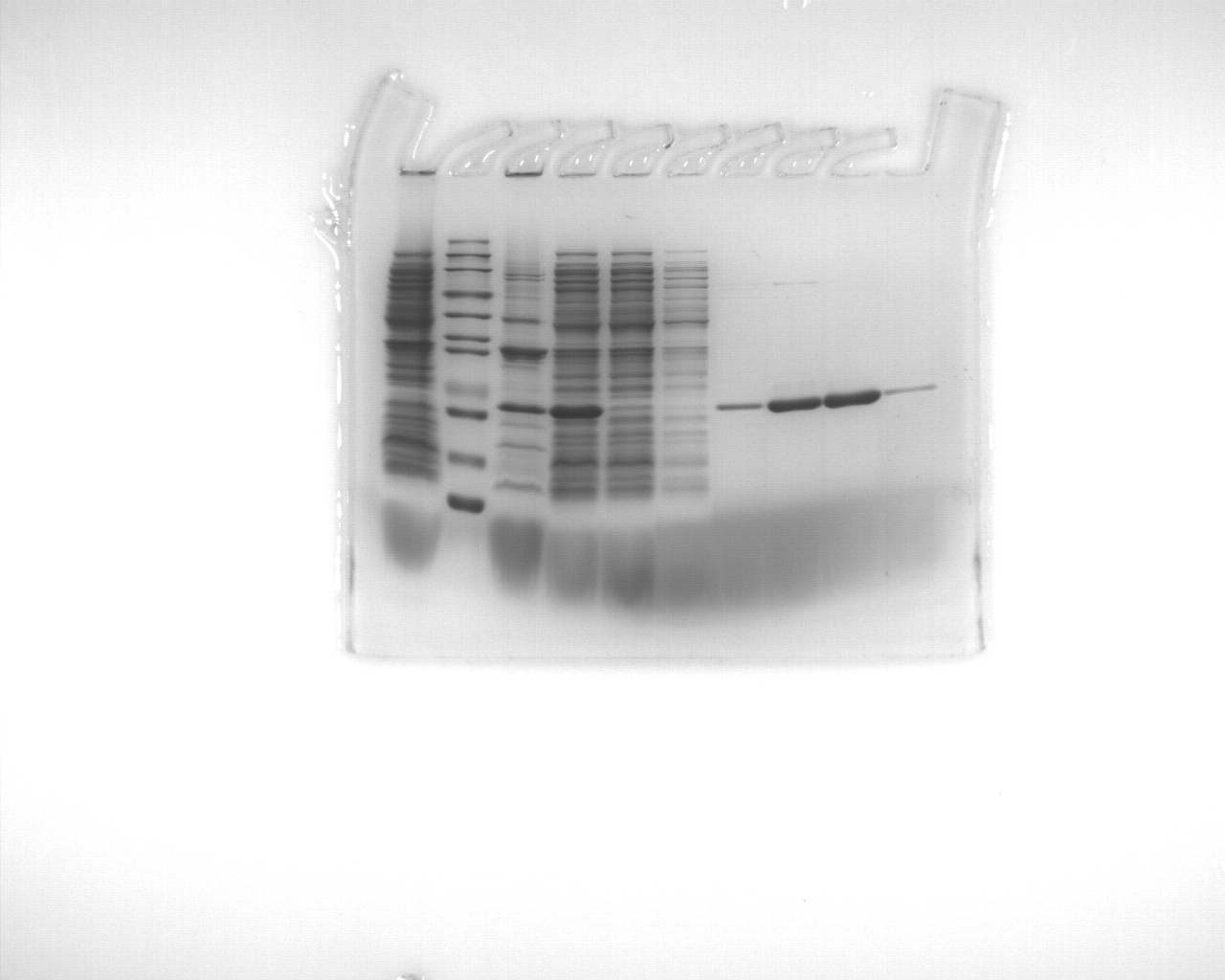

Supplement: Supplementary file 1 [file biology-15-00247-s001.zip › supplementary files - original images/original images of Figure S5.bmp]

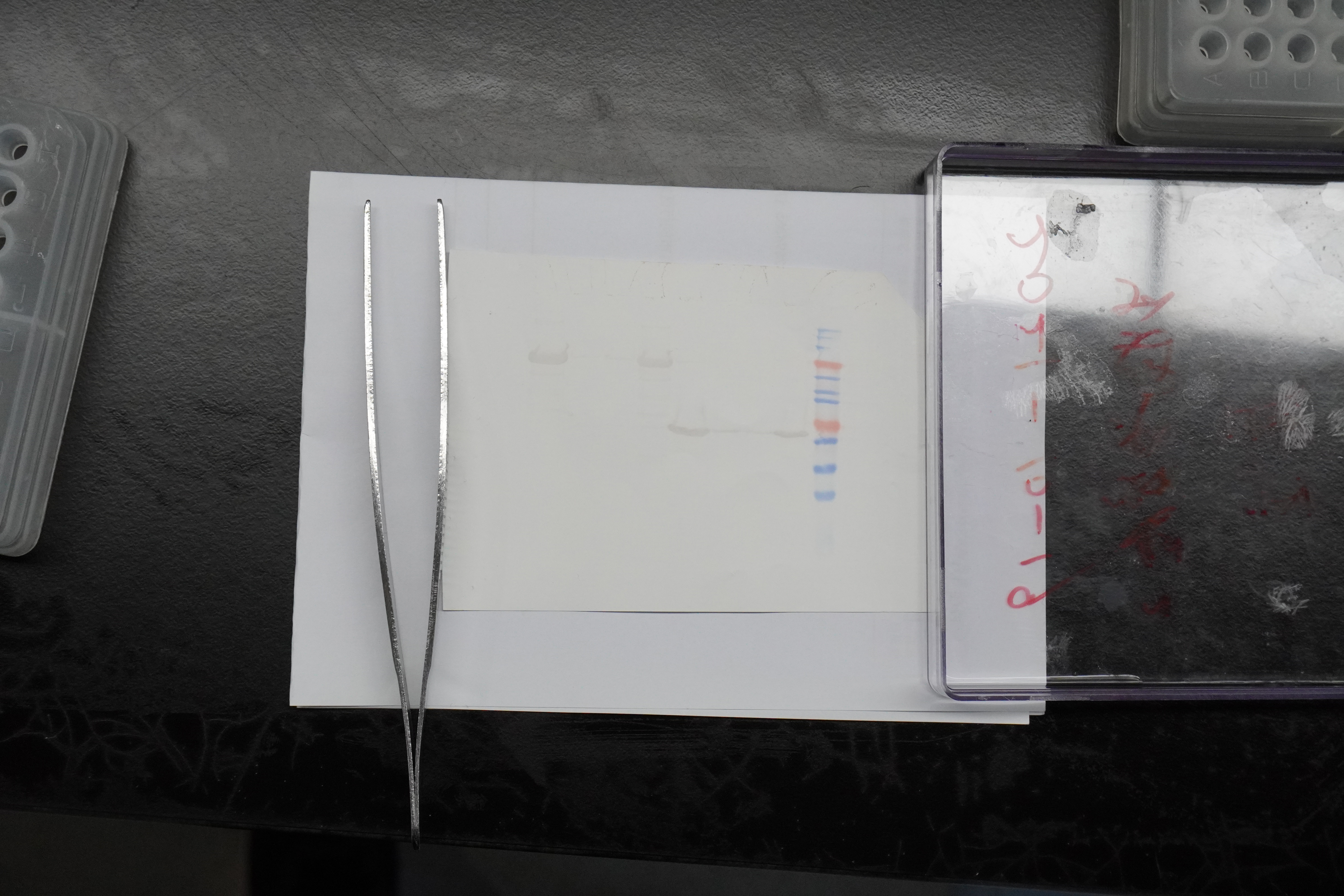

Supplement: Supplementary file 1 [file biology-15-00247-s001.zip › supplementary files - original images/original images of Figure S7.jpg]
